# Increased Mitotic Rate Coincident with Transient Telomere Lengthening Resulting from Pim-1 Overexpression in Cardiac Progenitor Cells

**DOI:** 10.1002/stem.1211

**Published:** 2012-08-22

**Authors:** Christopher T Cottage, Lauren Neidig, Balaji Sundararaman, Shabana Din, Anya Y Joyo, Brandi Bailey, Natalie Gude, Nirmala Hariharan, Mark A Sussman

**Affiliations:** San Diego State Heart Institute, San Diego State UniversitySan Diego, California, USA

**Keywords:** Pim-1, Telomere, Cardiac progenitor cell, Telomerase

## Abstract

Cardiac regeneration following myocardial infarction rests with the potential of c-kit+ cardiac progenitor cells (CPCs) to repopulate damaged myocardium. The ability of CPCs to reconstitute the heart is restricted by patient age and disease progression. Increasing CPC proliferation, telomere length, and survival will improve the ability of autologous CPCs to be successful in myocardial regeneration. Prior studies have demonstrated enhancement of myocardial regeneration by engineering CPCs to express Pim-1 kinase, but cellular and molecular mechanisms for Pim-1-mediated effects on CPCs remain obscure. We find CPCs rapidly expand following overexpression of cardioprotective kinase Pim-1 (CPCeP), however, increases in mitotic rate are short-lived as late passage CPCePs proliferate similar to control CPCs. Telomere elongation consistent with a young phenotype is observed following Pim-1 modification of CPCeP; in addition, telomere elongation coincides with increased telomerase expression and activity. Interestingly, telomere length and telomerase activity normalize after several rounds of passaging, consistent with the ability of Pim-1 to transiently increase mitosis without resultant oncogenic transformation. Accelerating mitosis in CPCeP without immortalization represents a novel strategy to expand the CPC population in order to improve their therapeutic efficacy. Stem Cells*2012;30:2512–2522*

## INTRODUCTION

Regenerative medicine to combat the devastating loss of myocardial contractility resulting from myocardial infarction (MI) has demonstrated promising initial results [1]. Increased left ventricular ejection fraction, decreased infarct size, and increased hemodynamic function following adoptive transfer of autologous c-kit+ cardiac progenitor cells (CPCs) suggests that infusion of CPCs play a pivotal role in cardiac regeneration [1–3]. However, CPCs isolated from patients are heterogeneous in nature and suffer from consequential impairment rooted in the underlying cardiomyopathic disease of the donor. Specifically, MI survivors typically are elderly, suffer from chronic cardiomyopathies or diabetes and thus possess CPCs with compromised regenerative potential [4, 5]. Aged CPCs exhibit short telomeres, inactive telomerase (TERT), and impaired proliferation, thereby limiting replicative capacity and generation of the population required for effective cellular cardiomyogenic treatment. Strategies aimed at accelerating proliferation and extending replicative lifespan of CPCs will be essential to overcome inherent limitations of harvested patient CPC populations derived from weak, aged, or damaged myocardium.

Telomeres are a nucleoprotein complex at the ends of linear chromosomes consisting of several kilobases of 5′-TTAGGG-3′ DNA repeats and telomere associated proteins collectively known as the shelterin complex [6, 7]. Shelterin assembles tightly on the telomere to prevent chromosomal instability and inhibit DNA damage machinery [8, 9]. Repeated rounds of mitosis or endogenous oxidative stress successively diminishes telomeric repeats resulting in critically short telomeres [10]. Short telomeres can lead to chromosomal fusions, cellular senescence, apoptosis, or transformation [11–13]. Telomere homeostasis is maintained by TERT, the reverse transcriptase responsible for telomere elongation [14], together with an associated RNA component (Terc) [15]. TERT adds telomeric repeats after DNA replication has taken place [16, 17], but TERT activation can also be initiated at transcriptional and post-translational levels [18]. Serine/threonine kinases Akt and PKC positively regulate TERT, activating enzyme activity and increasing telomere length [19]. c-Myc, a transcription factor that regulates as many as 15% of all known human genes [14] also promotes *TERT* transcription leading to increased TERT protein levels and activity [14, 20].

Pim-1, a serine/threonine kinase, promotes cell proliferation and survival in conjunction with c-Myc [21–23]. Previous studies from our group demonstrated enhanced myocardial regeneration by genetically engineering CPCs to overexpress Pim-1 [2] but the underlying cellular and molecular basis of Pim-1-mediated effects upon CPCs remain obscure. Understanding the molecular basis for Pim-1 enhancement of CPC activity is essential to delineate the mechanistic basis of Pim-1 activity and determine the potential of Pim-1-modified CPCs for incorporation into protocols for augmented clinical treatment of heart failure. Findings presented in this study demonstrate telomere length is transiently increased in CPCs overexpressing Pim-1 (CPCeP) correlated to acceleration of mitotic rate and decreased cell cycle time. Telomeric lengthening and telomerase activity stimulated by Pim-1 is dependent upon c-Myc activation and protects CPCs from doxorubicin-induced telomere attrition. Revealing these underlying mechanisms of telomere preservation and acceleration of mitosis by Pim-1 offers exciting new potential therapeutic interventions for CPC-mediated cardiac regeneration in heart failure.

## MATERIALS AND METHODS

### CPC Isolation, Culture, and Transduction

CPCs were isolated from mouse hearts based on expression of c-kit as previously described [24, 25]. CPCs from friend leukemia virus B inbred strain (FVB) mouse hearts at passage 14 were plated (0.5 × 10^4^ cells per well) in six-well plates, transduced with lentivirus (multiplicity of infection = 10), expanded, and isolated by fluorescently activated cell sorting to enrich for GFP^+^ cells. Cells were then maintained and passaged in CPC media [24]. Passage numbers indicated throughout are passage number post-transduction.

### Telomere Length Measurements

Telomere length was analyzed by quantitative fluorescent in situ hybridization (Q-FISH) and confocal microscopy. PNA probe was purchased from DAKO (K5325, Glostrup, Denmark, http://www.dako.com) and used according to manufacturer's protocol on cells fixed with 3:1 methanol/ acetic acid fixative and dried on glass chamber slides. Telomere signal was acquired in each nucleus using Leica LCS confocal software. To control for interday variation, slides with defined amounts of fluorescence were utilized to acquire appropriate signal intensity. A minimum of 150 CPCs per group was analyzed.

### Telomeric Repeat Amplification Protocol (TRAP)

For whole cell TRAP assays, CPC plates were scraped in 3-[3-cholamidopropyl) dimethyl-ammonio]-1-propanesulfonate buffer and then centrifuged at 4°C. Protein concentration was determined using Bradford assay and then manufacturer's protocol was followed for reverse transcriptase polymerase chain reaction (S7701, Millipore, Billerica, MA, http://www.millipore.com). Each group was subjected to heat inactivation as negative control. TSR8 positive controls with known TERT activity were used to create standard curve and assign TRAP activity units. Results are averages of three independent experiments in triplicate ± SEM.

### Proximity Ligation Assay (PLA)

The PLA kit was purchased from Olink Biosciences, and the manufacturer protocol was followed (Uppsala, Sweden, http://www.olink.com). Briefly, primary antibodies were applied after an hour block in 10% horse serum and incubated overnight at 4°C. The following day the slides were washed, then plus and minus PLA probes were applied in blocking buffer and incubated at 37°C. Next the slides were treated with ligation solution for 30 minutes followed by 90 minutes of amplification solution all at 37°C. After three washes, slides were treated with Sytox Blue and coverslipped in Vecta-shield mounting medium.

### CyQUANT Assay

A total of 1,000 CPCs were plated and allowed to adhere to a 96-well dish. CyQUANT solution was added at indicated time points and measured according to manufacturer's protocol (Invitrogen, Carlsbad, CA, http://www.invitrogen.com, Catalog number C35006). A CyQUANT assay performed 4 hours after plating confirms equal adherence of CPCs regardless of Pim-1 modification (supporting information Fig. 1A).

### Statistical Analysis

All data are expressed as mean ± SEM. Statistical analysis was performed using Student's *t* test and ANOVA with Tukey's post hoc analysis as appropriate. Telomere length distribution significance was determined using Kolmogorov-Smirnov test. *p* values less than 0.05 are considered significant.

## RESULTS

### Mitotic Rate Increased by Pim-1 Expression in CPCeP

**Figure 1:**
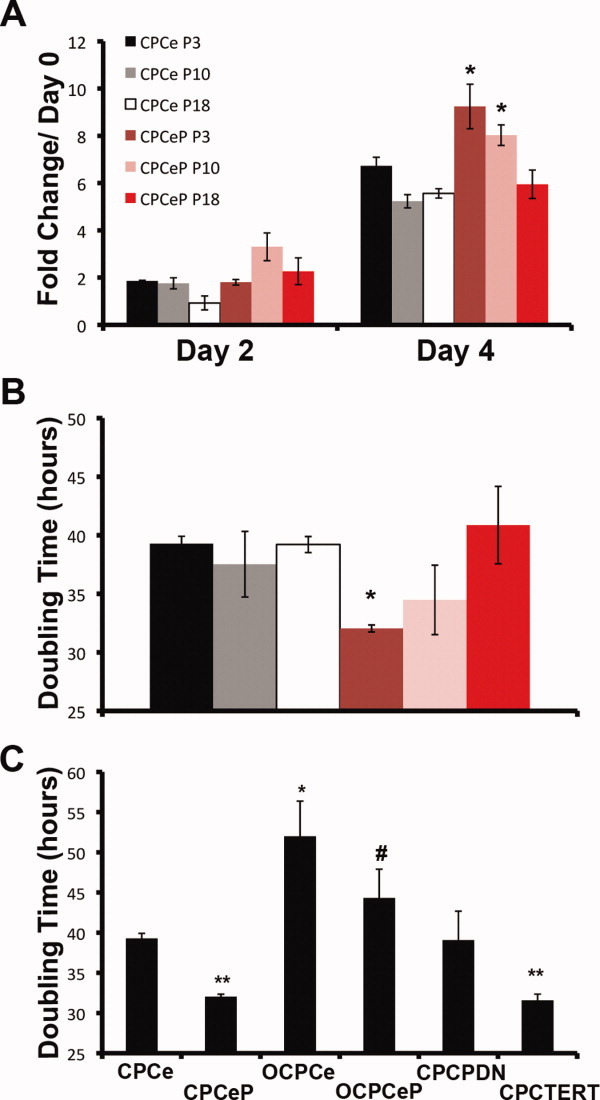
Temporary increases in proliferation and reduced CPC doubling time with Pim-1 overexpression. **(A):** CPC proliferation determined by CyQUANT assay. *, *p* < .05 versus CPCe at same passage. (**B, C):** Calculated CPC doubling time determined by CyQUANT and Trypan blue cell counts. **, *p* < .01 versus CPCe, #, *p* < .05 versus OCPCe. CPCe, CPCeP, CPCs isolated from 13-month old FVB mice overexpressing eGFP, Pim-1, CPCePDN, and CPCTERT. CPC doubling times in (C) are calculated three passages after transduction with indicated lentivirus. Abbreviations: CPC, cardiac progenitor cell; CPCe, CPCs overexpressing eGFP; CPCeP, CPCs overexpressing Pim-1; OCPCe, old CPCs overexpressing eGFP; OCPCeP, old CPCs overexpressing Pim-1; CPCPDN, CPCs overexpressing a kinase dead mutant of Pim-1; CPCTERT, CPCs overexpressing TERT.

Population proliferation in CPCeP early after transduction and subsequent passaging is increased 1.37-fold relative to that of control eGFP-expressing CPCs (CPCe) measured by CyQUANT DNA content assay. However, population proliferation rates of CPCePs equal that of CPCe by 18 passages postinfection (Fig. 1A). Increases in proliferation correlates to a significantly shorter doubling time in early passage CPCeP relative to CPCe (29.4 hours vs. 36.3 hours), but doubling time normalizes with CPCe at later passages (Fig. 1B). Pim-1 levels remain high throughout early and late passages (supporting information Fig. 2A) indicating that observed slowing of cell proliferation in late passage does not stem from diminished Pim-1 level.

Population doubling time is significantly increased 33% in CPCs isolated from hearts of 13-month-old mice (OCPC) relative to CPCs from 2-month old hearts (*p* = .02, Fig. 1C). Ability of Pim-1 expression to accelerate the mitotic clock in CPCs from aged hearts was assessed using old CPCs overexpressing Pim-1 (OCPCeP). Similar to findings with CPCeP, increased cellular proliferation of OCPCeP is evident relative to comparably prepared old eGFP-expressing CPC (OCPCe) controls measured by CyQUANT (supporting information Fig. 1; 11% and 29% increases at days 3 and 5, respectively). Enhanced OCPCeP proliferation that correlates with population doubling time significantly decreased by 15% compared to OCPCe (Fig. 1C). Rate of cell cycling for CPCs is not increased by overexpression of Pim-1 dominant negative (CPCePDN) mutant that lacks kinase activity, indicating that kinase activity is necessary for decreased doubling time. The increased mitotic rate of CPCeP is comparable to that observed with CPCs engineered to overexpress TERT, suggesting potential connection between Pim-1 overexpression and enhancement of TERT activity (Fig. 1C).

### Telomere Elongation in CPCs with Short Telomeres

**Figure 2:**
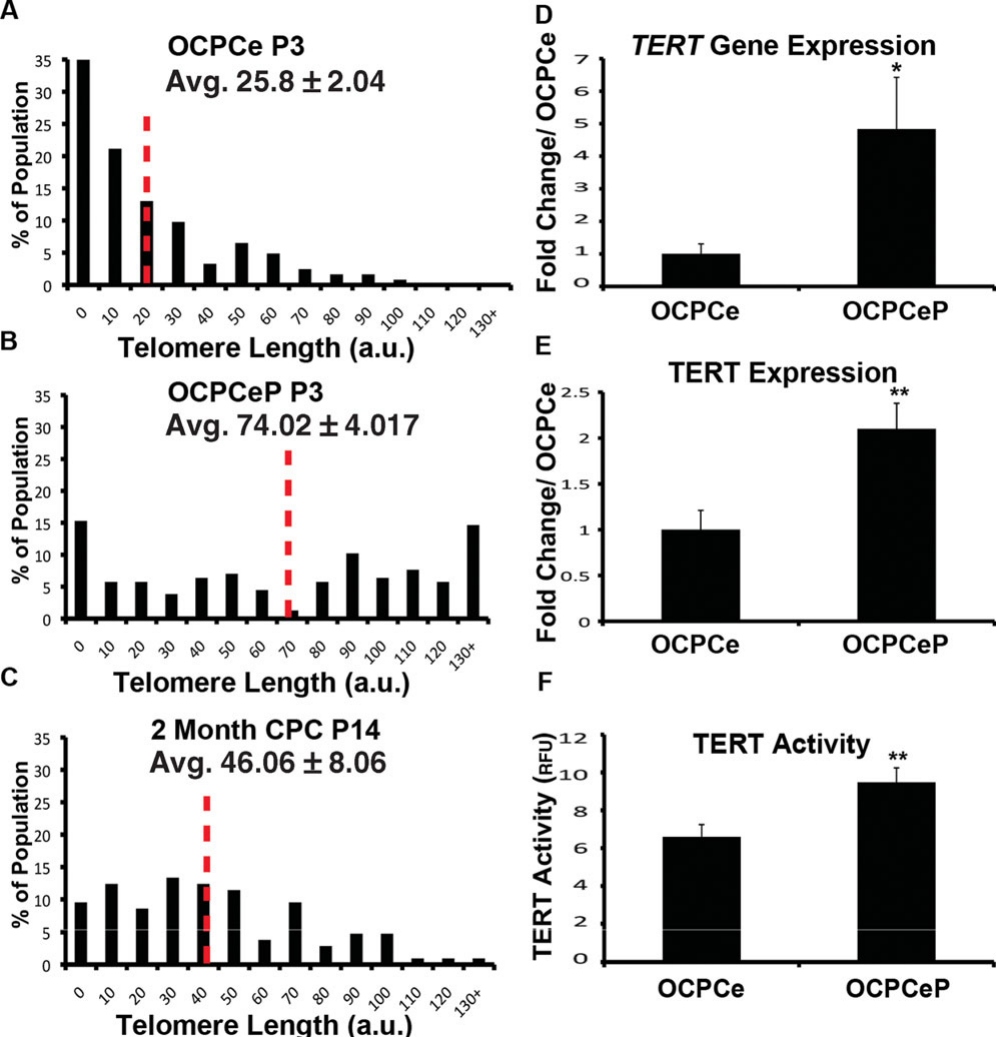
Telomerase activation and telomere lengthening in CPCs from aged hearts. **(A, B):** CPCs isolated from 13-month old mouse hearts and infected with eGFP or Pim-1 (OCPCe or OCPCeP, respectively) were plated and subjected to quantitative fluorescent in situ hybridization to determine telomere length (dashed red lines indicate averages). **(C):** Telomere lengths of CPCs isolated from 2-month-old FVB hearts demonstrate telomere lengths in younger CPCs. **(D):***TERT* gene determined by reverse transcription polymerase chain reaction. **(E):** TERT expression levels in OCPCe and OCPCeP three passages after infection. **(F):** TERT activity measured by TRAP assay. Dashed red lines represent average telomere lengths. Results are mean ± SEM. *, *p*, < .05 versus OCPCe; **, *p* < .01 versus OCPCe, telomere lengths between OCPCe and OCPCeP are significantly different *p* < .001 measured by KS test. Abbreviations: CPC, cardiac progenitor cell; OCPCe, old CPCs overexpressing eGFP; OCPCeP, old CPCs overexpressing Pim-1.

TERT activity regulates CPC growth and telomere length [26], so telomere length was compared between OCPCe and OCPCeP by QFISH. For example, OCPCe have an average telomere length of 25.8 relative fluorescent units (RFUs, Fig. 2A). Average RFUs of telomeres from OCPCeP are 2.9-fold higher than those of OCPCe indicative of telomere extension by Pim-1 kinase expression (Fig. 2A, 2B). In comparison, the average RFUs for CPCe versus OCPCe is 1.78 higher, confirming relatively shorter telomere length in OCPCe (Fig. 2C). Telomeric shortening is antagonized in aged mice by TERT overexpression [27], and increased levels of *TERT* gene, TERT protein, and TERT activity are observed in OCPCeP relative to OCPCe (Fig. 2D–2F, supporting information Fig. 2), providing a molecular basis for the effect of Pim-1 upon telomere length.

### Acute Telomere Lengthening in CPCeP Normalizes Through Culture Passages

**Figure 3:**
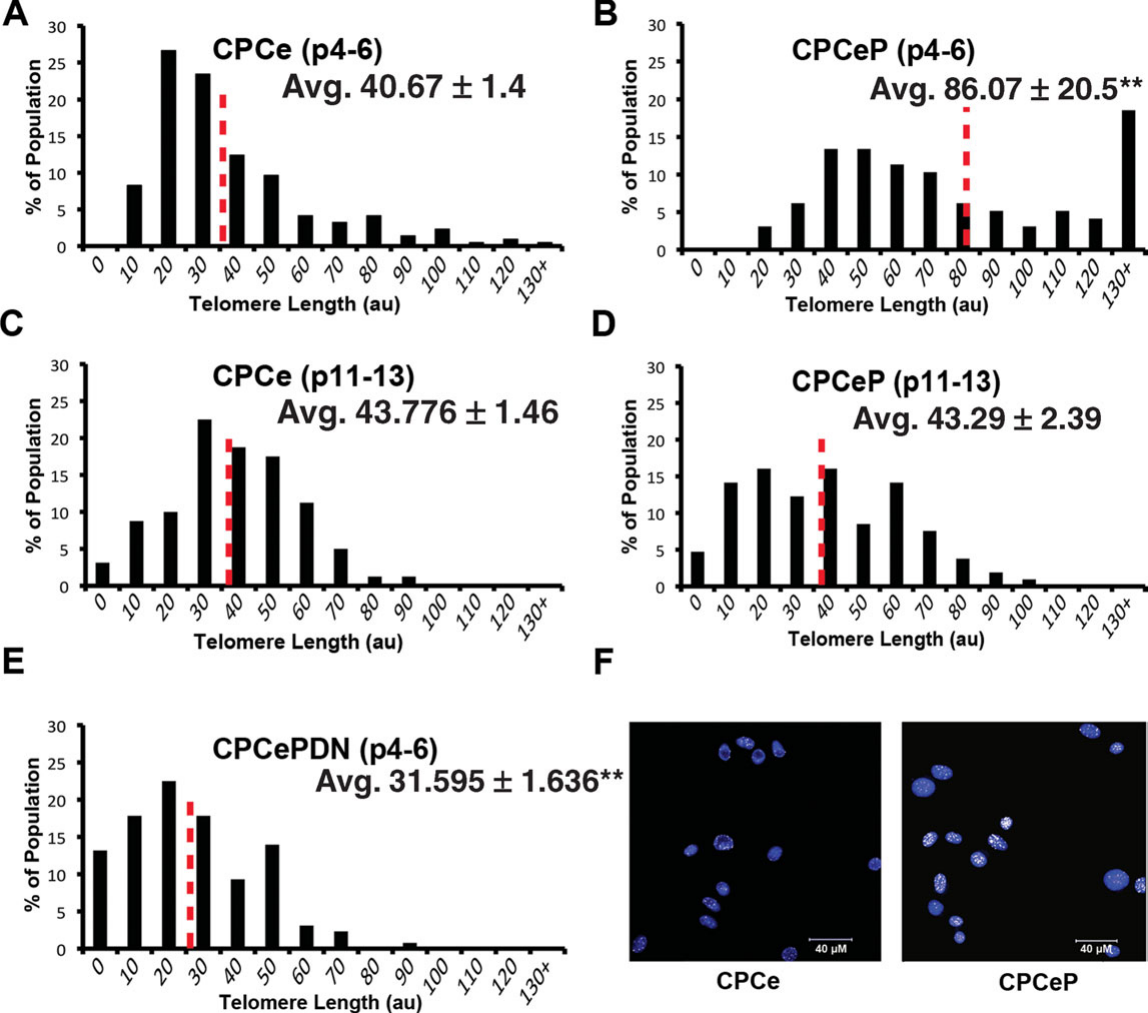
Telomere length is increased in CPCeP. **(A, B):** Telomere lengths in early passages (4–6) CPCe and CPCeP measured by quantitative fluorescent in situ hybridization (**, *p* < .01). **(C, D):** Telomere lengths in later passages (11–13) of CPCe and CPCeP, telomere lengths are no longer statistically different. **(E):** Telomere length is significantly decreased in early passage CPCePDN (**, *p* < .01 vs. CPCe by KS test). Dashed red lines represent average telomere lengths. **(F):** CPCe and CPCeP telomeres visualized by QFISH (white foci), nuclei are stained using To-Pro-3 (blue). Abbreviations: CPC, cardiac progenitor cell; CPCe, CPCs overexpressing eGFP; CPCeP, CPCs overexpressing Pim-1; CPCePDN, CPCs overexpressing a kinase dead mutant of Pim-1.

Increases in mitotic rate (Fig. 1) and telomere length (Fig. 2) are evident soon after Pim-1 overexpression in cultured CPCs, so persistence of these enhancements was subsequently determined by serial passaging of CPCeP versus CPCe in culture.comparative telomere measurements based upon RFUs show a significant increase of 2.1-fold for CPCeP versus CPCe from passages 4-6 (Fig. 3A, 3F; *p* < .01) demonstrating that Pim-1 can increase telomere length even in CPCs derived from hearts of young mice. However, the increased RFUs detected for CPCeP telomere length is lost with continued serial passage, with comparable signal between CPCeP and CPCe by passages 11-12 in culture (Fig. 3C, 3D). Participation of Pim-1 in preservation of telomere length is evident in CPCePDN that show significant 23% loss of RFUs at early passages (*p* < .01, Fig. 3E). Telomere extension depends upon TERT activity since CPCeP treated with TERT inhibitor MST 312 show no increase in RFUs compared to CPCe (supporting information Fig. 3). Collectively, these results establish Pim-1 as a mediator of TERT activity that can promote transient telomere elongation early after cell modification that correlates temporally with increased mitotic activity.

### Telomere Extension in CPCeP Depends Upon Increased TERT Activity

**Figure 4:**
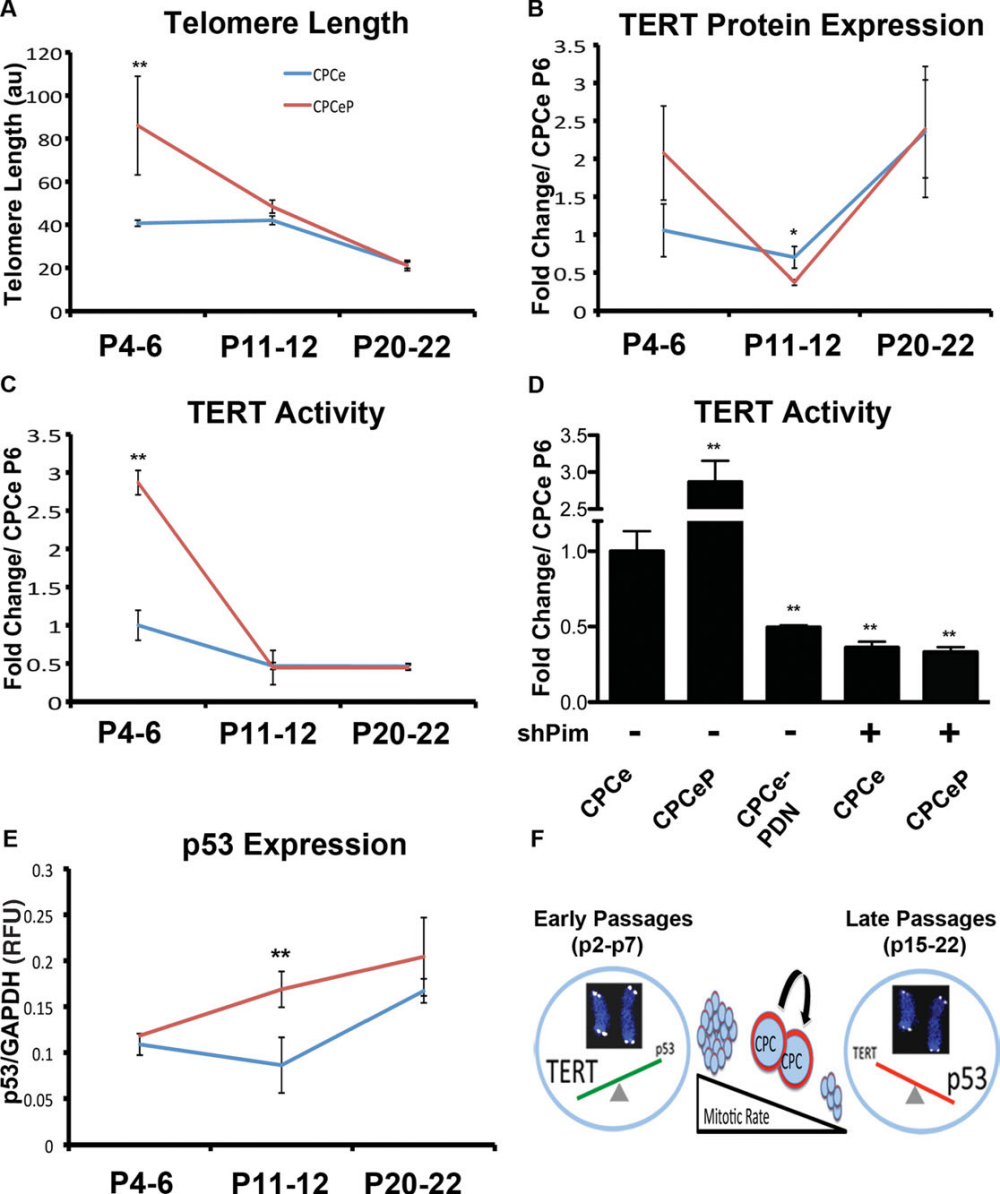
Telomere lengthening in CPCeP is due to acute increases TERT activity. **(A):** Average telomere lengths in CPCe and CPCeP throughout 22 passages, **, *p* < .001 versus CPCe passages 4–6. **(B):** TERT protein expression at varying passages, values are fold change divided by low passage CPCe (4–6). **(C):** Telomerase activity of CPCe and CPCeP from various passages post-transduction. **(D):** Telomerase activity in low passage CPCePDN (p 4–6) and after short hairpin deletion of Pim-1 in CPCe and CPCeP. **(E):** p53 protein expression at multiple passages determined by immunoblot and standardized to GAPDH expression. **(F):** Summary of mechanism for transient telomere lengthening and increased mitotic rate in CPCeP. **, *p* < .01; *, *p* < .05 all statistics determined by KS test, Student's *t* test, or two-way ANOVA where applicable. Abbreviations: CPC, cardiac progenitor cell; CPCe, CPCs overexpressing eGFP; CPCeP, CPCs overexpressing Pim-1; CPCe-PDN, CPCs overexpressing a kinase dead mutant of Pim-1; shPim, short hairpin deletion of Pim-1.

Telomere average length determined by RFUs from serial passage of CPCeP shows early augmentation at passages 4-6 that subsequently normalizes to that of CPCe by passages 11-12 and is maintained comparably between CPCeP and CPCe until passages 20-22, which is considered late passage (Fig. 4A). Whereas the acute telomere lengthening in early passage CPCs correlates with significant increases in TERT activity (2.99-fold), this elevation of TERT is not present in CPCe, CPCePDN, or mid to late passage CPCeP (Fig. 4D; supporting information Fig. 2A). Specifically, TERT protein levels decrease 5.54-fold in CPCeP at passages 11-12 relative to early passage coincident with telomere length normalization. Again, TERT activity is augmented by Pim-1 kinase as demonstrated by significant reductions resulting from knockdown using short hairpin lentivirus for Pim-1 in both CPCe and CPCeP (Fig. 4D). Tumor suppressor protein p53 levels are minimal and comparable to CPCe in early passages. However, p53 precipitously increases in CPCeP through serial passaging (42.6% more in p11-12 and 21.1% more in p20-22 vs. p11-12, Fig. 4E; supporting information Fig. 2C) consistent with decreases in telomere length and TERT activity in later passages. Taken together the data suggest that TERT is upregulated early in CPCeP leading to increased TERT activity and longer telomeres. This effect reverses with serial passaging resulting in increased p53, reduced TERT, a slower rate of mitosis, and diminished telomeres (Fig. 4F).

### TERT Interacts with Pim-1

**Figure 5:**
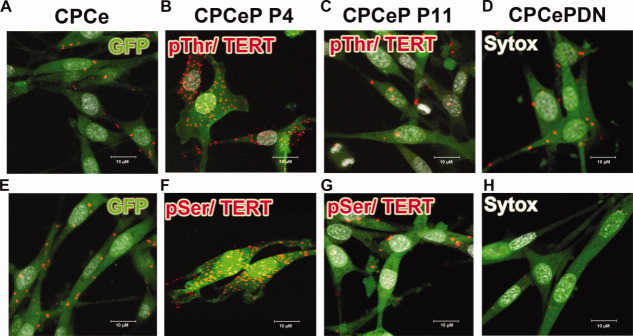
TERT phosphorylation declines over passaging in CPCeP. **(A–D):** Phosphorylated TERT on threonine residues and serine residues (**E–H**, red), cell body is visualized using GFP (green), and nuclei are stained with Sytox (white). Early passage CPCeP (4) demonstrates increased pThr/TERT and pSer/TERT that diminishes over passaging (CPCeP passage 11). Abbreviations: CPC, cardiac progenitor cell; CPCe, CPCs overexpressing eGFP; CPCeP, CPCs overexpressing Pim-1; CPCePDN, CPCs overexpressing a kinase dead mutant of Pim-1; GFP, green fluorescent protein.

Pim-1 and TERT interact directly as assessed by coimmunoprecipitation of whole cell lysates at passage 7 (supporting information Fig. 4A, 4B). Interaction is independent of kinase activity as TERT was also pulled down by immunoprecipitation with CPCePDN (supporting information Fig. 4A, 4B). Localization of Pim-1/TERT interactions was determined by proximity ligation assays (PLAs) performed using CPCe or CPCeP. TERT and Pim-1 interaction is evident in all three cell types, with the highest level of interaction evident in CPCeP (supporting information Fig. 4A, red dots). Pim-1/TERT PLA signal is absent in control CPCeP samples lacking primary antibody as well as dramatically reduced by Pim-1 kinase inhibitor Quercetagetin (Q) or silencing RNA specific to Pim-1 (siPim-1; supporting information Fig. 4C–4H). TERT phosphorylation could occur on several possible serine and threonine residues by Pim-1 [28, 29], but specific residues for pSer-TERT or pThr-TERT have not been identified. Demonstration of pSer-TERT or pThr-TERT using antibodies to TERT and phospho-threonine or phospho-serine by PLA signal shows abundant pThr-TERT signal in CPCe, CPCeP, and CPCePDN (Fig. 5A–5D) with PLA signal for pThr-TERT in CPCeP diminishing by passage 11 (Fig. 5B, 5C). Similarly, pSer-TERT PLA signal is observed throughout the cell, but diminished pSer-TERT PLA signal in CPCePDN suggests Pim-1-mediated serine phosphorylation. PLA results for pSer-TERT or pThr-TERT were corroborated by diminution of signal upon introduction of pharmacologic inhibition using Q or silencing using siPim-1 (supporting information Fig. 4D, 4G). Collectively, decreased telomere length, diminished TERT activity, together with reduced PLA signal for Pim-1/TERT and pSer/TERT in CPCePDN point to Pim-1/TERT interaction with serine residue phosphorylation augmenting TERT activation and telomere elongation.

### c-Myc Inhibition Blocks Pim-1 Telomere Preservation

**Figure 6:**
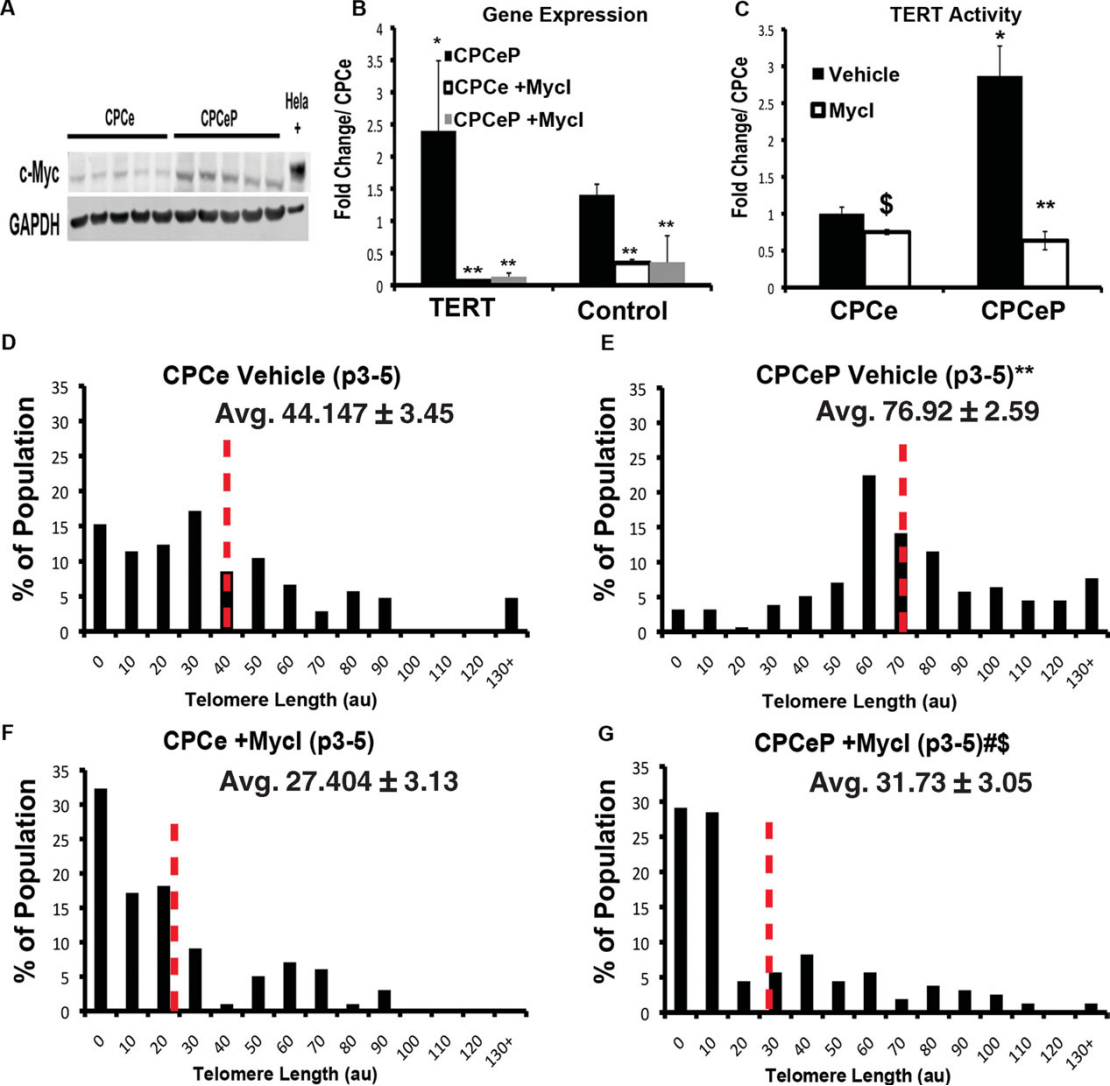
Myc inhibition blocks Pim-1 mediated telomere elongation. **(A):** Expression of c-Myc by immunoblot, GAPDH is used as a loading control. **(B):** Reverse transcription polymerase chain reaction of c-Myc target genes TERT and eLF4 (control) in vehicle (DMSO) CPCeP or CPCe and CPCeP +MycI (50 μM for three passages) values are fold change over vehicle treated CPCe. **(C):** TERT activity in vehicle and MycI CPCe and CPCeP (*, *p* < .01 vs. CPCe vehicle treated; **, *p* < .01 vs. CPCeP vehicle treated; ^$^, *p* < .05 CPCe vehicle vs. CPCe MycI treated). **(D, E):** Telomere lengths of vehicle-treated CPCe and CPCeP and (**F, G**) Myc I treated, telomere length determined by quantitative fluorescent in situ hybridization (**, *p* < .0001 vs. vehicle CPCe; ^#^, *p* = .002 vs. MycI CPCe, and ^$^, *p* < .0001 vs. CPCeP vehicle determined by KS test). Abbreviations: CPC, cardiac progenitor cell; CPCe, CPCs overexpressing eGFP; CPCeP, CPCs overexpressing Pim-1.

Synergistic interaction between c-Myc and Pim-1 that promotes CPC proliferation and survival [21, 22] implicates a role for c-Myc in the molecular mechanism of Pim-1 action. Indeed, c-Myc expression is increased 4.4-fold in early passages 4-7 CPCeP (Fig. 6A). c-Myc inhibition with the small molecule 10058-F4 (MycI, 50 μM) results in significant depression for mRNA levels of *TERT* the c-Myc target *eLF4* in low passage CPCe and CPCeP (Fig. 6B). TERT activity activated by c-Myc [30] is significantly decreased by MycI, with greater reduction evident in CPCeP relative to CPCe (Fig. 6C, 4.52-fold decrease). Continued presence of MycI leads to significant telomere length diminution in CPCeP relative to vehicle-treated controls, with similar effects also present in CPCe (Fig. 6G, 6F) indicating a pivotal role for c-Myc in Pim-1-induced telomere maintenance.

### Telomere Length Preservation After Doxorubicin

**Figure 7:**
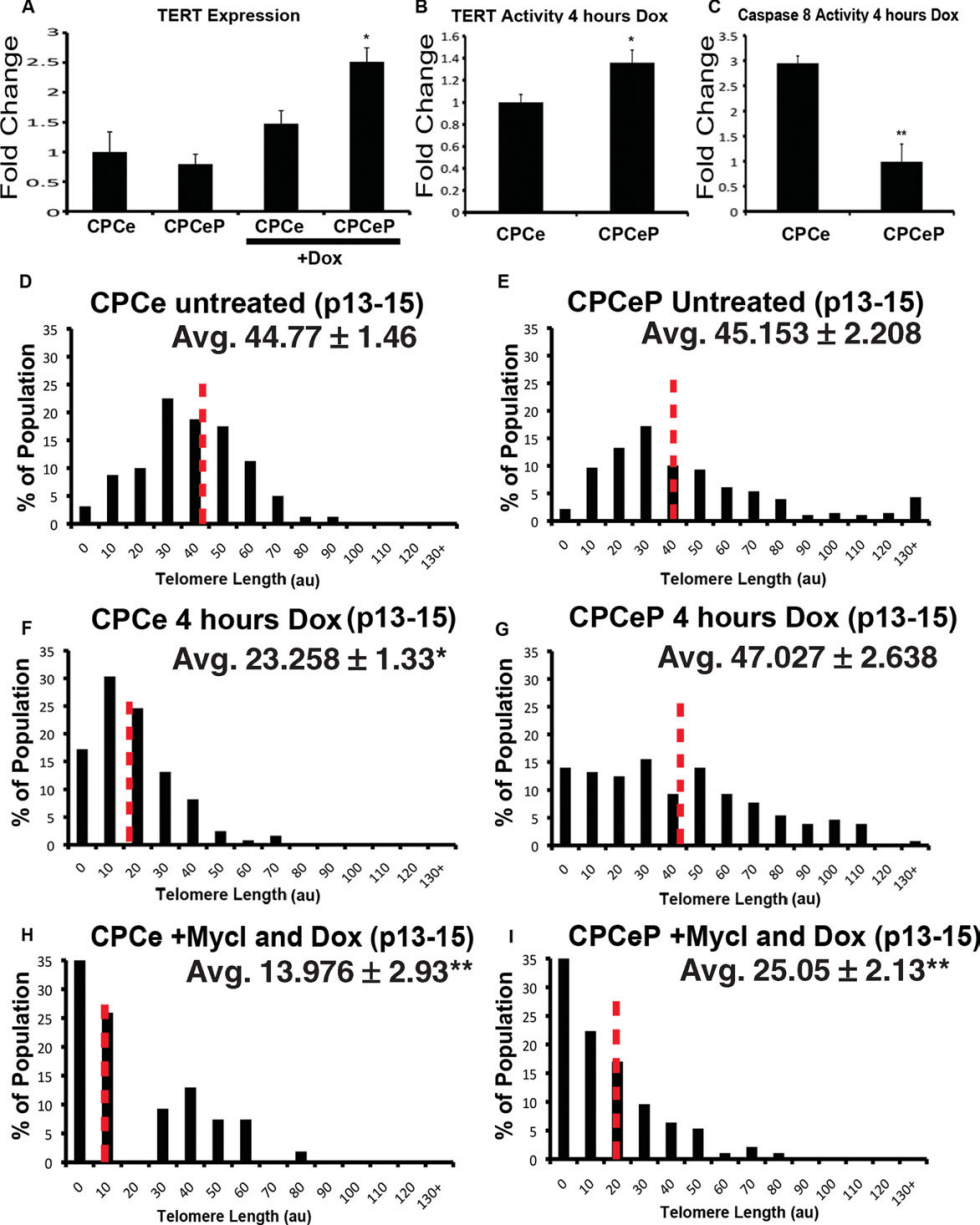
Telomere length is preserved in CPCePs challenged with Doxorubicin. **(A):** Fold change increase in TERT protein in CPCeP after Dox treatment (passages 13–15, 4 hours 1 μM Dox). **(B):** TERT activity in CPCs 4 hours after Dox measured by TRAP, values are fold change over Dox treated CPCe (*, *p* < .05). **(C):** Caspase eight activity was determined after Dox treatment; values are fold change over untreated CPCe. **(D–G):** QFISH analysis in untreated and Dox treated CPCe and CPCeP at the same passages. **(H, I):** Telomere length measurements in CPCe and CPCeP treated with Dox and MycI. (*, *p* < .01 vs. untreated CPCe; **, *p* < .001 vs. untreated CPCe). Abbreviations: CPC, cardiac progenitor cell; CPCe, CPCs overexpressing eGFP; CPCeP, CPCs overexpressing Pim-1; Dox, Doxorubicin.

Doxorubicin (Dox), a cardiotoxic anthracycline, depletes CPCs in the myocardium through oxidative stress [31], and oxidative stress mediated by Dox leads to telomeric shortening [32]. Therefore, ability of Pim-1 to antagonize Dox-induced telomeric shortening in CPC was tested at passages 13-15 when telomere length is comparable between CPCe and CPCeP. TERT expression increases in response to Dox exposure, but challenged CPCeP exhibit increases of 1.70-fold in TERT protein and 1.28-fold in activity relative to comparably treated CPCe (Fig. 7A, 7B; *p* < .05). Telomere length is maintained following Dox challenge in CPCeP, whereas telomere attrition is observed in CPCe (Fig. 7D–7G). Similar to previous findings (Fig. 6), telomere preservation in Dox-challenged CPCeP depends upon c-Myc activity, as introduction of MycI abrogates telomere length preservation in CPCeP (Fig. 7H, 7I). Dox-induced apoptosis was increased 2.98-fold in CPCe relative to vehicle-treated controls measured by caspase 8 activity. In contrast, Dox failed to increase caspase 8 activity in CPCeP relative to vehicle-treated control cells (Fig. 7C). DNA synthesis as measured by bromodeoxyuridine (BrdU) incorporation is comparable over the 4-hour treatment period in either CPCe or CPCeP. Increased BrdU incorporation likely due to increased DNA damage [32, 33] as a result of Dox challenge is observed in both CPCe and CPCeP (supporting information Fig. 5B). Collectively, these results demonstrate the ability of Pim-1 to resist the detrimental effects of doxorubicin treatment on CPCs.

## DISCUSSION

Current excitement related to clinical implementation of regenerative medicine is tempered by limitations in efficiency of stem cell-mediated repair, including low efficiency engraftment, persistence, and expansion of adoptively transferred cell populations. A clinical trial infusing autologous CPCs in patients suffering from MI damage with ejection fraction <40% shows partial recovery of myocardial structure and function, but the fate of donated cells cannot be readily assessed [1]. Furthermore, patient age and disease progression are likely to compromise proliferative potential and survival of CPCs in the setting of regenerative therapy. Thus strategies aimed at extending cellular replicative lifespan may be essential to reinvigorate CPCs derived from aged or chronically stressed cardiac tissue samples to reconstitute the damaged myocardium. Extrapolating interpretations from experimental animal MI models ranging from mice to pigs treated with autologous stem cells reveal consistent benefits in myocardial structure and function despite significant losses of donated cells shortly after adoptive transfer [34–36]. However, molecular interventions to enhance survival and persistence of adoptively transferred stem cells improve restoration of myocardial performance and reverse pathologic damage relative to control cells alone [37, 38]. Specifically, genetic modification of CPCs with Pim-1 expression leads to superior cellular regeneration while concurrently increasing cell numbers necessary to improve myocardial structure and function as shown using either murine or human derived CPCs [2, 3]. Telomere length of CPCeP engrafted into damaged myocardium was found to be significantly longer by 2.02- and 2.34-fold relative to CPCe using either murine- [2, 3] or human-derived [2, 3] CPCs, respectively. In addition, CPCeP exhibit a twofold increase in asymmetric chromatid segregation together with enhanced proliferation, thereby increasing the number of committed cells available for myocardial repair [39]. Results presented here are consistent with the premise that myocardial Pim-1 expression prolongs the proliferative phase of CPC growth resulting in a hyperplastic phenotype [22]. The mechanistic basis for beneficial effects of Pim-1 engineering likely rests, in part, with the capacity for proliferation coupled with maintenance of telomere length, both characteristics of youthful myocardial cells. Collectively, antagonism of CPC aging phenotype together with enhanced survival signaling make Pim-1 an attractive molecular interventional approach to enhance CPC-mediated myocardial regeneration.

Precedents in highly mitotic cells support critical roles for c-Myc in acceleration of proliferation. Immortalization of human and mouse fibroblasts occurs with ectopic expression of c-Myc in the presence of activated Ras, however, overexpression of c-Myc alone increases cellular proliferation without transformation [40, 41]. CDC25a, a CDK activating phosphatase and target of c-Myc promotes G1/S transition by targeting and activating cyclin/CDK2 complexes [42, 43]. Interestingly, fibroblasts enter S-phase 2–3 hours faster when cells overexpress CDC25a, demonstrating that CDC25a is sufficient to stimulate DNA synthesis [43]. G1/S transitions are promoted through Pim-1-dependent increases in CDC25a activity and the inhibitory actions of p21 phosphorylation [44]. Pim-1 synergistically co-operates with c-Myc to promote proliferation in neoplastic cell lines and CPCs [22, 45]. Future studies will aim to elucidate the relationship between Pim-1 and CDC25a in CPC mitotic clock regulation.

Dysfunctional telomeres signal cell senescence and cell cycle arrest, however, mitosis can be rescued through reactivation of TERT [46]. Shortened telomeres limit proliferation of cells, slowing the mitotic clock that in time cripples the regenerative capacity of tissues [47]. Senescent CPCs are characterized by short telomeres, increased p53, cell cycle arrest, and heart failure due to the inability of CPCs to replenish apoptotic cardiomyocytes [26, 48]. TERT knockout mice suffer from decreased cardiomyocyte mitosis, increased p53 induced apoptosis, and dilated cardiomyopathy [49] consistent with hearts that have exhausted their CPC reserve. Telomere lengthening due to enhanced TERT activity delays the aging process by allowing more rounds of progenitor cell mitosis and increasing the longevity of the animal [50]. The molecular basis for our results is consistent with the postulate that transient telomere elongation and accelerated cell cycle hinge on the expression of TERT and p53 throughout passaging, as c-Myc and Pim-1 overexpression remain constant in CPCeP (Fig. 4F). Shortly after CPCs are transduced with Pim-1, TERT is upregulated and activated thereby lengthening telomeres. Concurrently Pim-1 drives mitosis and diminishes population doubling time. Acute cell cycle rate increases and telomere lengthening normalizes as TERT activity and expression levels decrease and p53 levels increase. Elevated p53 expression prompted in response to diminished telomere length slows mitosis, thereby inhibiting CPC immortalization. Engineering CPCs ex vivo with Pim-1 to activate TERT and stimulate mitosis is an attractive means of opposing the deleterious effects of aging that is not perpetual and does not lead to cellular immortalization.

Cardiomyopathy in patients treated with doxorubicin is postulated to result from depletion of the CPC population that, in turn, diminishes replacement of damaged myocytes leading to ventricular dilation, wall thinning, and premature mortality [31, 32]. Mechanistically, doxorubicin acts as an oxidative stressor creating DNA damage and promoting apoptosis [51]. Variability in cardiotoxic response to doxorubicin treatment among patients may be a consequence of individual differences in telomere length and TERT activity in the CPCs. Telomere attrition produced by doxorubicin administration is blunted in CPCeP, offering a novel molecular therapeutic intervention for doxorubicin-induced cardiotoxic damage as well as enhancement of regenerative potential for CPC derived from aged or chronically stressed myocardium.

In conclusion, as clinical trials using autologous progenitor cell transplantation advance, the quality of progenitor cells administered is of critical importance. The cellular and molecular phenotypes of the CPCs will determine their efficacy in cardiac repair. Results from this study suggest that Pim-1 promotes a short-lived increase in mitotic rate in conjunction with an acute lengthening of CPC telomeres, characteristics of youthful CPCs. Augmenting the regenerative potential of CPCs through molecular engineering with Pim-1 enhances proliferation and antagonizes the phenotypic characteristics of aging, providing a mechanistic basis for observations of enhanced regeneration using CPCeP in the damaged myocardium.

## CONCLUSIONS

- Increased rate of mitosis in CPCeP.
- Acute transient telomere lengthening in CPCeP.
- Pim-1-dependent telomere lengthening requires C-Myc activity in CPCs.
- Telomeric shortening induced by Dox can be blunted by Pim-1.

## Supplementary material

Supplemental Figure 1. Increased population cycling in early passage CPCeP and CPCs isolated from 13 month old hearts and infected with Pim-1, related to Figure 1. A. Cyquant assay performed on CPCe and CPCeP passage 5 (P5) and measured at 4, 6, 24, and 30 hours after plating. B. Cyquant assay performed on isolated older CPCs infected with lentivirus overexpressing GFP (OCPCe) or Pim-1 (OCPCeP). #p=0.09 vs. OCPCe day 3, *p<0.05 vs. OCPCe day 5.

Supplemental Figure 2. Western blots from quantitations in text, related to Figures 2 and 4. A-C. Immunoblots of CPCe and CPCeP at varying passages and probed using antibodies against the indicated proteins. + indicates whole lysates from Hela cells, e is CPCe and eP is CPCeP. ++ Indicates whole cell lysates from CPCs infected with lentivirus overexpressing TERT

Supplement Figure 3. Telomere lengthening in CPCeP is dependent on TERT activity, related to Figure 3. A-D. Early passage CPCs treated with either DMSO or telomerase inhibitor (MST-312, 1μM).

Supplemental Figure 4. Pim-1 interacts with TERT in CPCePs, related to Figure 5. A. Pim-1 and TERT interactions demonstrated by PLA for Pim-1 and TERT (red). B. Coimmunoprecipitation of Pim-1 and TERT. C PLA performed on CPCeP without primary antibodies; this control was performed with every assay to confirm specific staining. D and E. PLAs of Pim-1 and TERT (red), Pim-1 and pS/pT and Tert (red) in CPCeP treated with Pim-1 inhibitor, Quercetagetin (Q, 10μM for 24 hours). F. Pim-1 expression knocked down with a Pim-1 specific siRNA. Lane 1 is CPCeP, lane 2 is CPCe+ siPim- 1, and lane 3 is CPCeP +siPim-1. G and H. PLAs of Pim-1 and TERT (red) and pS/pT and Tert CPCeP treated with siPim-1.

Supplemental Figure 5. TERT expression and DNA incorporation after Dox treatment, related to Figure 7. A. Immunoblots of CPCe and CPCeP treated with Dox and probed for indicated antibodies + indicates whole cell Hela lysates. B. DNA synthesis measured by BrdU incorporation in DOX treated and untreated CPCs. $ p<0.05 vs untreated CPCe.
