# Supplementary figures and images for: Increased Mitotic Rate Coincident with Transient Telomere Lengthening Resulting from Pim-1 Overexpression in Cardiac Progenitor Cells
Source: Stem Cells. 2012 Aug 22;30(11):2512–22. doi: 10.1002/stem.1211 (PMC3479348; doi:10.1002/stem.1211)

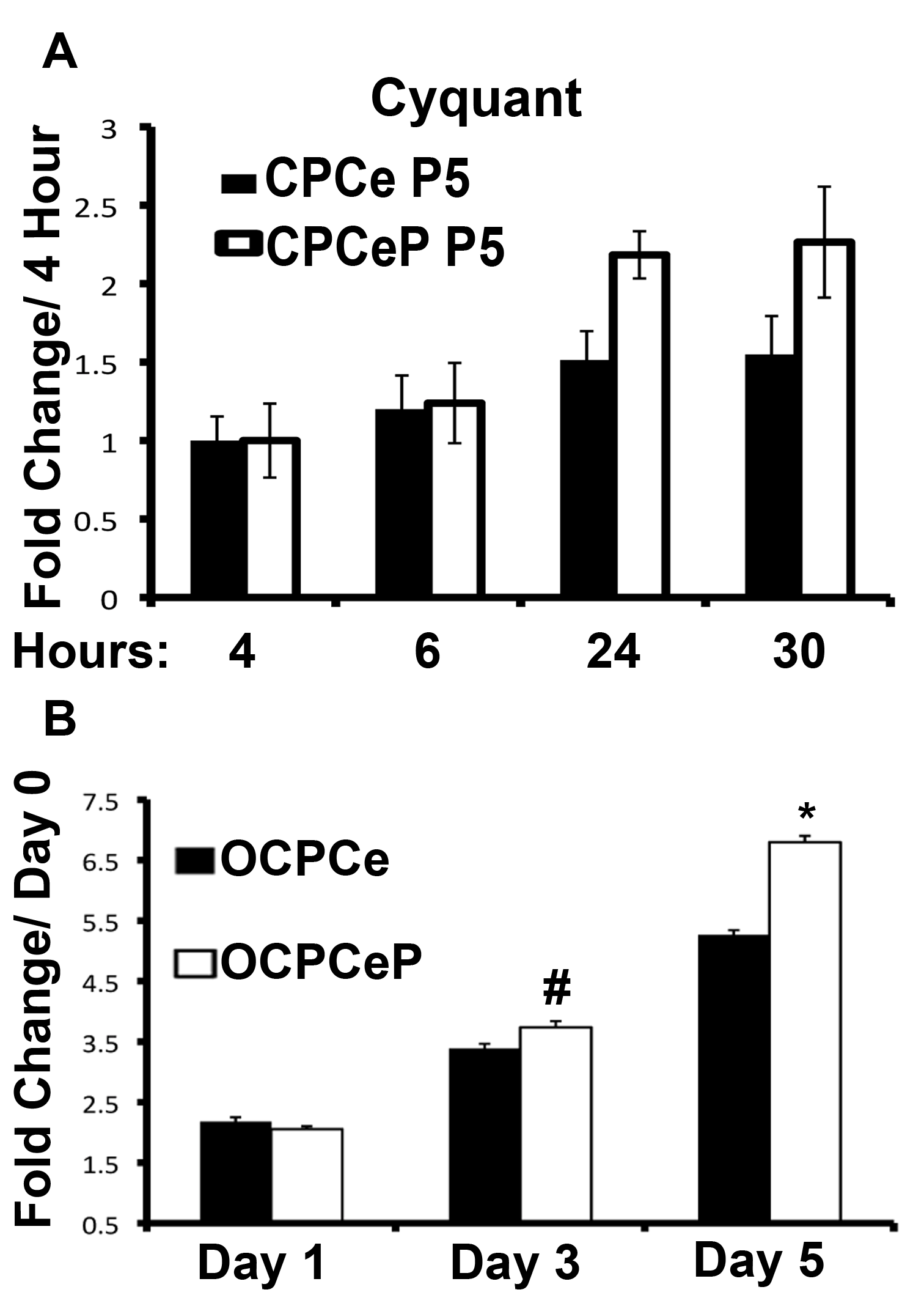

Supplement: Supplementary file 1 [file stem0030-2512-SD1.tif]

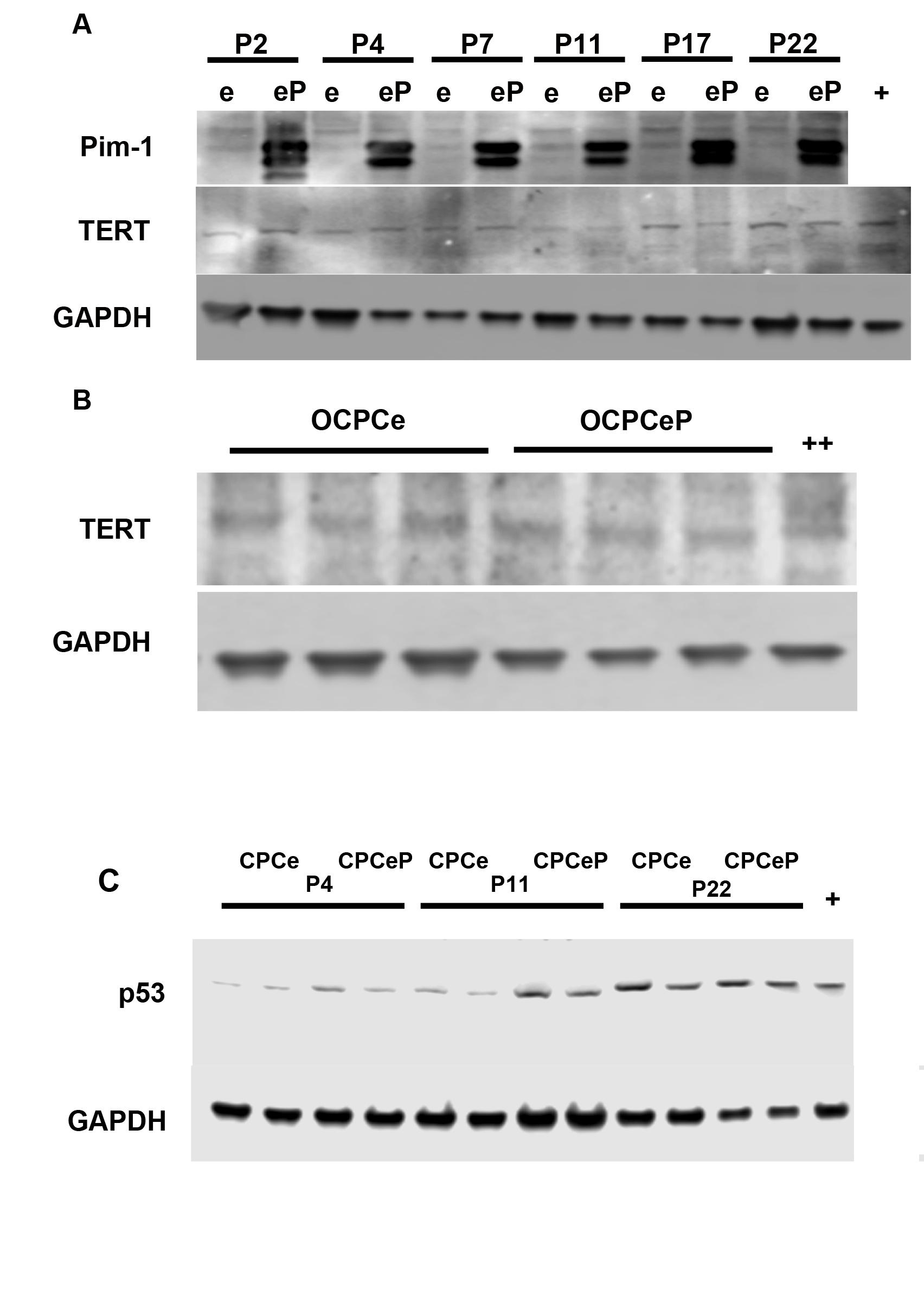

Supplement: Supplementary file 2 [file stem0030-2512-SD2.tif]

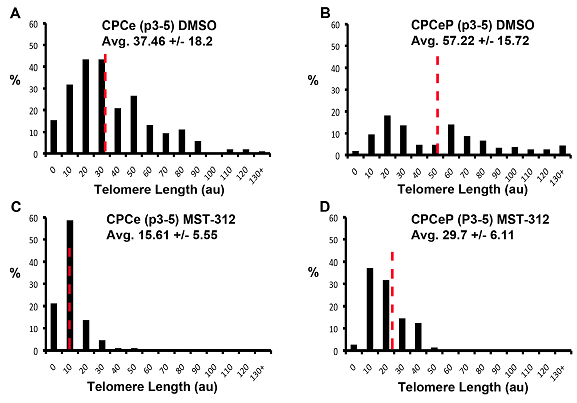

Supplement: Supplementary file 3 [file stem0030-2512-SD3.tif]

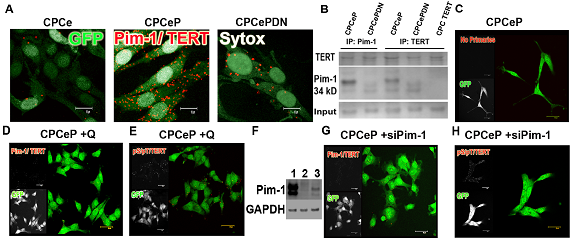

Supplement: Supplementary file 4 [file stem0030-2512-SD4.tif]

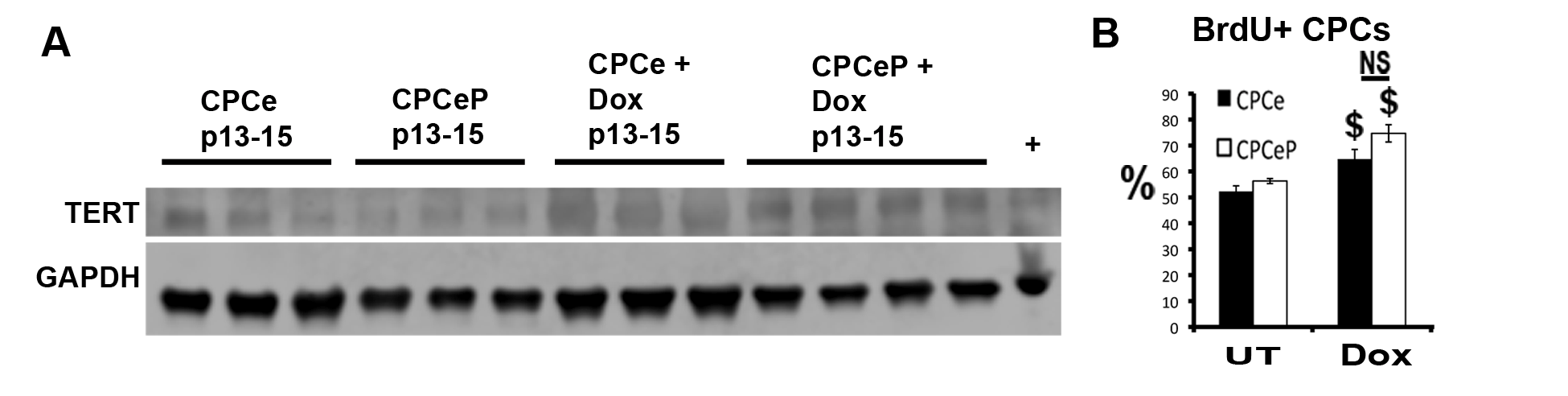

Supplement: Supplementary file 5 [file stem0030-2512-SD5.tif]
