# Supplementary material for: Increased Mitotic Rate Coincident with Transient Telomere Lengthening Resulting from Pim-1 Overexpression in Cardiac Progenitor Cells
Source: Stem Cells. 2012 Aug 22;30(11):2512–22. doi: 10.1002/stem.1211 (PMC3479348; doi:10.1002/stem.1211)
Supplement: Supplementary file 6 [file stem0030-2512-SD6.pdf]

Supplemental Materials and Methods

Real Time PCR. Total RNA was isolated from CPCs using Quick RNA MiniPrep (Zymo Research) and reverse-transcribed to cDNA using iScript cDNA Synthesis Kit (Biorad). RT-PCR was performed in duplicate using iQ SyBR Green (Biorad) according to the manufacturer's protocol. Data are standardized to 18S ribosomal RNA and are the averages of three experiments +/- S.E.M., primer sequences are provided in Supplemental Table 1.

Immunocytochemistry CPCs on 2 chamber glass slides were fixed in 4% paraformaldehyde then washed 3 times in PBS. Cells were permeablized with 0.1M Glycine 0.2% Triton X for 2 minutes followed by 3 washes in PBS. Cells were then blocked in 10% horse serum/PBS for 1 hour, and then primary antibodies were applied in blocking buffer. A section accompanied all stainings without primary antibody as a negative control.

Doxorubicin Treatment, Pharmacologic Inhibitors, Caspase 8, and Cell Cycling. CPCs were treated for 4 hours with 1 μmol/L of Dox in CPC growth media. Small molecule c-Myc inhibitor 10058-F4 (Sigma, 50μM) was administered to CPCs and continually passaged in the presence of inhibitor for indicated passages. MST 312, telomerase inhibitor (Sigma, 1 μM) was administered to CPCs and continually passaged in the presence of inhibitor for indicated passages. CPC apoptosis was determined by Caspase 8 activity (Clontech Laboratories, APT129). CPC proliferation was determined by BrdU incorporation, one hour before the end of the experiment CPCs were incubated with BrdU (10 μM) then fixed in 4% paraformaldehyde. DNA synthesis was determined by BrdU incorporation, one hour before the end of the experiment CPCs was incubated with BrdU (10 μM) then fixed in formaldehyde. Doubling time was calculated using the following formula DT = log N/log 2, where N is the cell number of the harvested CPCs at the end of the experiment divided by the initial number of cells seeded.

Immunoblot Analysis Whole cell lysates isolated from CPCs were prepared in 1X SDS sample buffer (22). Lysates were sonicated briefly then boiled for 5 minutes and used or stored at -80 C. Samples were loaded into an Invitrogen 4-12% Tris-Glycine mini-gel and run at 150V for 1.5 hours on an Invitrogen electrophoresis apparatus. Separated proteins were transferred to a PVDF membrane pre-incubated in methanol then blocked for 1 hour with 5% dry non-fat milk in TBS-T (50 mmol/liter Tris-HCl (pH 7.6)/150 mM NaCl/ 0.1% Tween 20). After transfer, the membrane was probed with primary antibodies overnight at 4°C with gentle agitation in blocking buffer. Primary antibodies include: TERT (Abcam, ab5181) and GAPDH (Chemicon). The next day blots were washed with TBS-T three times and probed with fluorescent, HRP, or alkaline phosphatase-conjugated secondary antibodies 1:1000 in blocking solution (Jackson Labs) for 2 hours at room temperature. Following three washes with TBS-T blots were scanned using a Typhoon 9410 (GE Healthcare) and signal quantitated using NIH Image J software.

Supplemental Data

Gene	Forward Primer 5'-3'	Reverse Primer 5'-3'
TERT	TGAACACCAGCCCACCGCAC	GGTCCTTCCCCGGGCTGCT
eLF4	GGACGGGATTGAGCCTATGTG	CAGCAGTGTCTCTAGCCAGAAG
Pim-1	GCGGCTTCGGCTCGGTCTACT	TGCCATTAGGCAGCTCTCCCCA

Table 1. Primers used in Study.

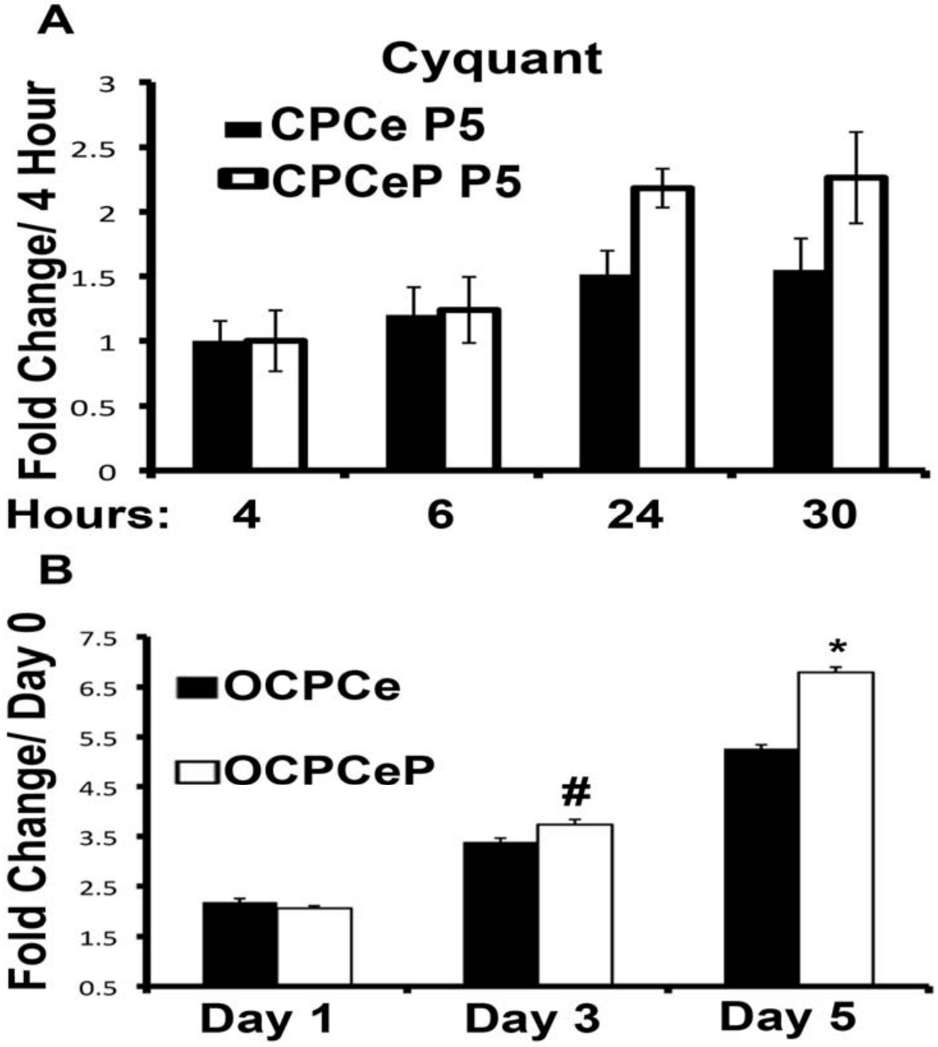

Supplemental Figure 1. Increased population cycling in early passage CPCeP and CPCs isolated from 13 month old hearts and infected with Pim-1, related to Figure 1. A. Cyquant assay performed on CPCe and CPCeP passage 5 (P5) and measured at 4, 6, 24, and 30 hours after plating. **B.** Cyquant assay performed on isolated older CPCs infected with lentivirus overexpressing GFP (OCPCe) or Pim-1 (OCPCeP). #p=0.09 vs. OCPCe day 3, *p<0.05 vs. OCPCe day 5.

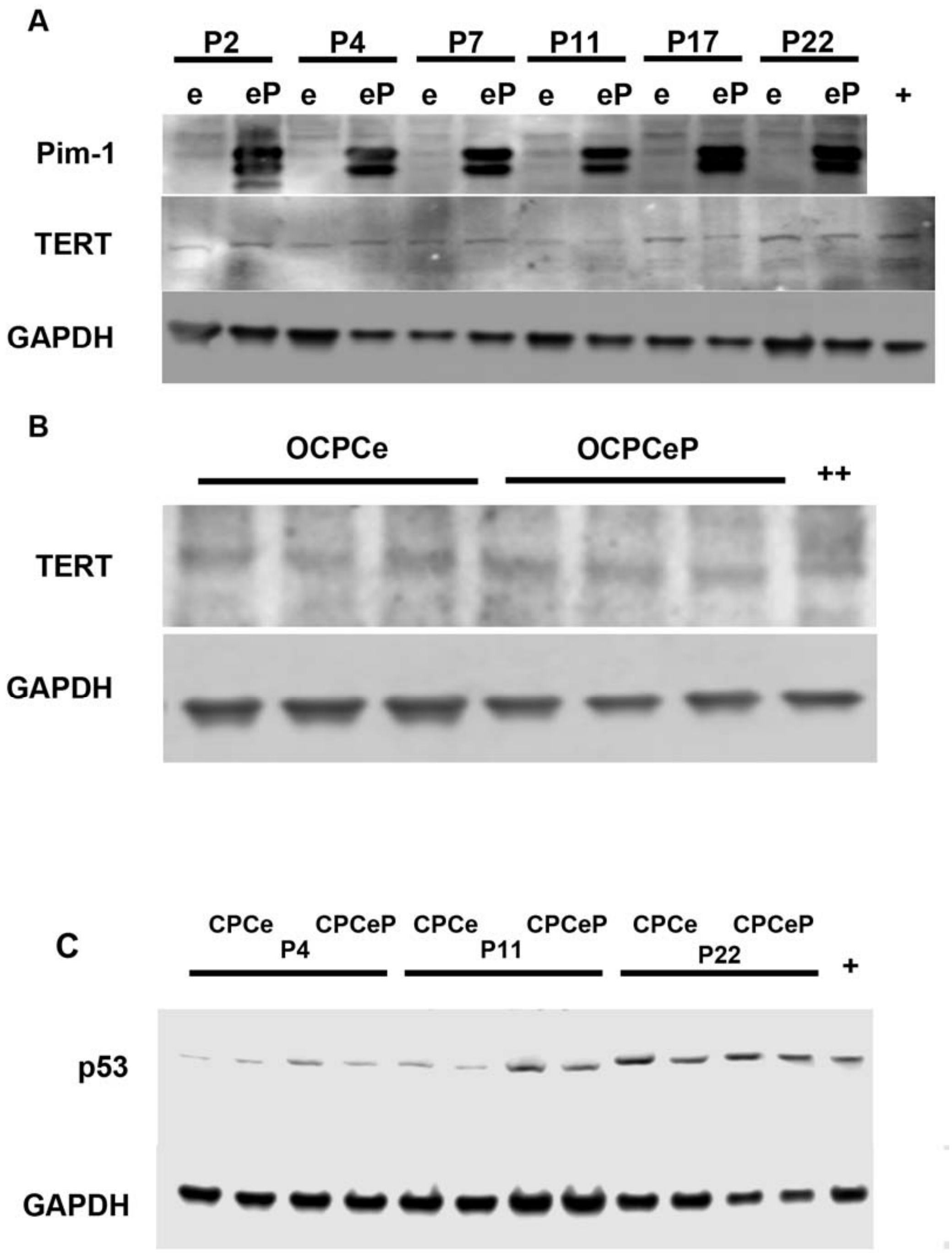

Supplemental Figure 2. Western blots from quantitations in text, related to Figures 2 and 4. A-C. Immunoblots of CPCe and CPCeP at varying passages and probed using antibodies against the indicated proteins. + indicates whole lysates from Hela cells, e is CPCe and eP is CPCeP. ++ Indicates whole cell lysates from CPCs infected with lentivirus overexpressing TERT

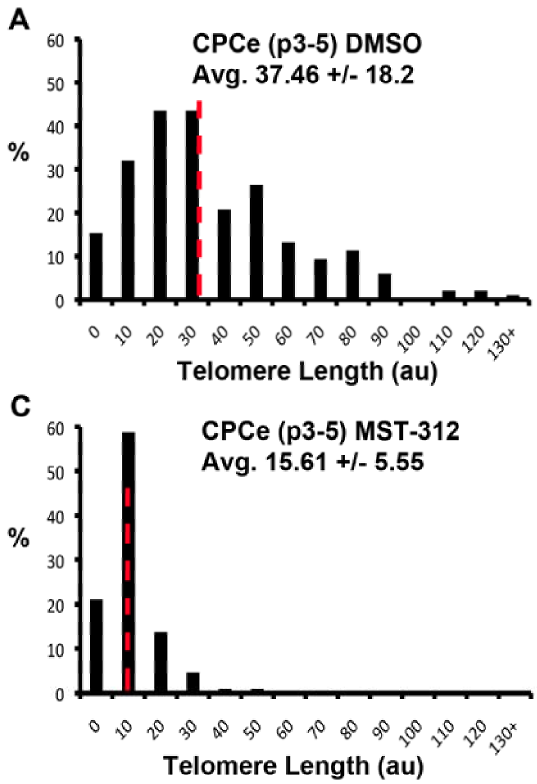

Supplement Figure 3. Telomere lengthening in CPCeP is dependent on TERT activity, related to Figure 3. A-D. Early passage CPCs treated with either DMSO or telomerase inhibitor (MST-312, 1μM).

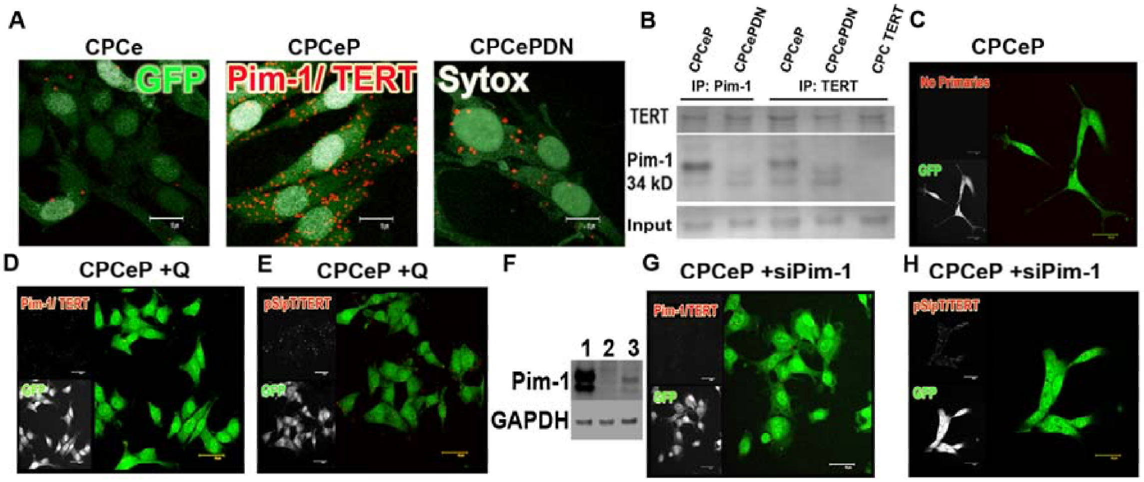

Supplemental Figure 4. Pim-1 interacts with TERT in CPCePs, related to Figure 5. A. Pim-1 and TERT interactions demonstrated by PLA for Pim-1 and TERT (red). **B.** Co-immunoprecipitation of Pim-1 and TERT. **C** PLA performed on CPCeP without primary antibodies; this control was performed with every assay to confirm specific staining. **D and E.** PLAs of Pim-1 and TERT (red), Pim-1 and pS/pT and Tert (red) in CPCeP treated with Pim-1 inhibitor, Quercetagetin (Q, 10μM for 24 hours). **F.** Pim-1 expression knocked down with a Pim-1 specific siRNA. Lane 1 is CPCeP, lane 2 is CPCe+ siPim-1, and lane 3 is CPCeP +siPim-1. **G and H.** PLAs of Pim-1 and TERT (red) and pS/pT and Tert CPCeP treated with siPim-1.

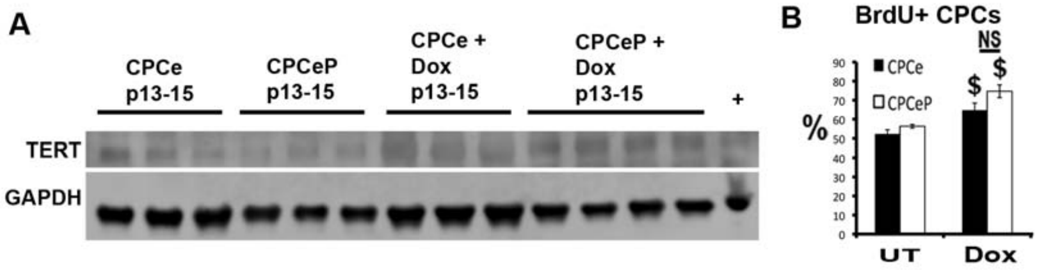

Supplemental Figure 5. TERT expression and DNA incorporation after Dox treatment, related to Figure 7. A. Immunoblots of CPCe and CPCeP treated with Dox and probed for indicated antibodies + indicates whole cell Hela lysates. **B.** DNA synthesis measured by BrdU incorporation in DOX treated and untreated CPCs. $ p<0.05 vs untreated CPCe.
